# The Effects of Remote Ischemic Preconditioning and N-Acetylcysteine with Remote Ischemic Preconditioning in Rat Hepatic Ischemia Reperfusion Injury Model

**DOI:** 10.1155/2014/892704

**Published:** 2014-01-08

**Authors:** Ali Ihsan Uysal, Elvan Ocmen, Mert Akan, Sevda Ozkardesler, Bekir Ugur Ergur, Ensari Guneli, Tuncay Kume, Uğur Koca, Belgin Unal Togrul

**Affiliations:** ^1^Department of Anesthesiology and Reanimation, Dokuz Eylul University Medical School, Inciralti, 35340 Izmir, Turkey; ^2^Department of Histology and Embryology, University Medical School, 35340 Izmir, Turkey; ^3^Department of Laboratory Animal Science, University Medical School, 35340 Izmir, Turkey; ^4^Department of Biochemistry, University Medical School, 35340 Izmir, Turkey; ^5^Department of Public Health, University Medical School, 35340 Izmir, Turkey

## Abstract

*Background*. Remote ischemic preconditioning (RIP) and pharmacological preconditioning are the effective methods that can be used to prevent ischemia reperfusion (IR) injury. The aim of this study was to evaluate the effects of RIP and N-Acetylcysteine (NAC) with RIP in the rat hepatic IR injury model. *Materials and Methods*. 28 rats were divided into 4 groups. Group I (sham): only laparotomy was performed. Group II (IR): following 30 minutes of hepatic pedicle occlusion, 4 hours of reperfusion was performed. Group III (RIP + IR): following 3 cycles of RIP, hepatic IR was performed. Group IV (RIP + NAC + IR): following RIP and intraperitoneal administration of NAC (150 mg/kg), hepatic IR was performed. All the rats were sacrificed after blood samples were taken for the measurements of aspartate aminotransferase (AST) and alanine aminotransferase (ALT) levels and liver was processed for conventional histopathology. *Results*. The hepatic histopathological injury scores of RIP + IR and RIP + NAC + IR groups were significantly lower than IR group (*P* = 0.006, *P* = 0.003, resp.). There were no significant differences in AST and ALT values between the IR, RIP + IR, and RIP + NAC + IR groups. *Conclusions*. In the present study, it was demonstrated histopathologically that RIP and RIP + NAC decreased hepatic IR injury significantly.

## 1. Introduction

Liver ischemia/reperfusion injury (IRI) may occur during surgery, like hepatectomy or transplantation, or systemic hypoxia, like respiratory or circulatory failure. Reperfusion can cause more damage than ischemia itself [1]. During ischemia toxic oxygen radicals are produced in the tissues. These oxygen and superoxide radicals can cause endothelial injury, an increase in microvascular permeability and tissue edema in the reperfusion period [2, 3]. Besides activated adhesion molecules and cytokines can start systemic inflammatory response, which are known as IRI [3]. Although tissue ischemia is the main starter of the pathophysiological changes, reperfusion causes inflammation [4].

Liver IRI can cause hepatic failure especially in the presence of coexisting hepatic disease [5] or even multiple organ failure [6–8].

Ischemic preconditioning (IP) is one of the most common techniques that are used to reduce hepatic IRI [9]. After a short period of ischemia/reperfusion, prolonged ischemia reperfusion (IR) causes less injury in the tissue, which is called direct IP [10]. Similarly, brief ischemia/reperfusion stimulus to an organ can protect a remote organ against IRI and this is called remote ischemic preconditioning (RIP) [11]. Pharmacological method is another way to protect the tissue from IRI and many different drugs were studied for this purpose [9, 12]. N-Acetylcysteine (NAC) is one of the most commonly used drugs in several IRI studies [13–17].

The aim of this study is to compare the effectiveness of RIP and NAC addition to RIP in the rat hepatic IRI model.

## 2. Material and Methods

After approval of the Experimental Animal Research Committee of our institution, the study was conducted at the experimental animals' laboratory of our institute. Twenty-eight adult male Wistar albino rats weighing 250–300 g were used in this study.

Rats were randomized into four groups: Group I (sham, *n* = 7): following laparotomy, at the 65th minute of the anesthesia hepatic pedicle dissection was performed and waited till the 270th minute of the anesthesia without any procedure. Group II (IR, *n* = 7): At the 65th minute of the anesthesia, total hepatic ischemia for 30 minutes and four hours of reperfusion were performed. Group III (RIP + IR, *n* = 7): following laparotomy, three cycles of RIP applied to left hind limb were performed 5 minutes before hepatic IR. Group IV (RIP + NAC + IR, *n* = 7): 150 mg/kg NAC (*Asist amp *300 mg/3 mL* amp, Hüsnü Arsan İlaç Sanayi, Turkey*) was administered intraperitoneally at the 60th minute of the anesthesia in addition to the procedures of group III.

### 2.1. Experimental Protocol

Rats were anesthetized with intraperitoneal 50 mg/kg ketamine (*Ketalar flk., Pfizer Pharma GMBH*, *Germany*) and 10 mg/kg xylazine hydrochloride (*Alfazyne *% 2, *Alfasan International, Holland*). Anesthesia was maintained with intraperitoneal 25 mg/kg ketamine when needed. Durations of anesthesia were equal in all groups.

Laparotomy was performed with a midline incision. After the liver and the hepatic pedicle were visualized a microvascular clamp was used for performing ischemia. Successful occlusion of the pedicle was confirmed by the change of the color of the liver. Rats were heated with a heating lamp during the operation to maintain a rectal body temperature of 37–37.5°C. Hydration was maintained with subcutaneous infusion of 3 mL/kg/h saline solution.

The effectiveness of the RIP method that we used has been shown previously [18, 19]. For this purpose an elastic bandage (1 cm width and 30 cm length) to the proximal left hind limb was wrapped circularly 3 times and squeezed. Three cycles of 10-minute ischemia and 10-minute reperfusion were performed.

At the end of the study, sternotomy was performed under anesthesia to all rats and blood samples were taken from the right atrium. Then hepatectomy was performed for histopathology and rats were sacrificed by exsanguination. Liver samples were fixed for 24–48 hours in 10% buffered formaldehyde and examined by a light microscope.

Congestion, necrosis, cytoplasmic vacuolization, cytoplasmic hypereosinophilia, nuclear pyknosis, and inflammatory cell number were examined to assess the degree of the liver injury. Hepatic histopathological injury score (HHIS) [20] was classified according to the severity of the injury (grade 0: minimal or no injury; grade 1: mild injury; grade 2: moderate injury; grade 3: severe injury).

Blood samples were centrifuged for 10 minutes at 3000 rpm and plasma samples were stored at −20°C until the measurement. *Cobas Integra 800, Roche, USA,* analyzer was used for the measurements of Aspartate aminotransferase (AST) and alanine aminotransferase (ALT) levels.

For statistical analysis, SPSS 15.0 (Statistical Package for the Social Sciences version 15, Chicago, IL, USA) was used. Kruskal-Wallis variance analysis was performed to analyze the data. All data were expressed as mean ± standard deviation (mean ± SD) using Mann-Whitney *U* test for pair wise comparisons of groups. The level of statistical significance was accepted as *P* < 0.05.

## 3. Results

A total of 28 rats were included in the study. None of the rats died during the study period.

### 3.1. Hepatic Histopathological Injury Scores

The HHIS of the sham operated group was significantly lower than the IR, RIP + IR, and RIP + NAC + IR groups (resp., *P* = 0.001, *P* = 0.001, *P* = 0.002). The scores were significantly higher in the IR group than in the RIP + IR, and RIP + NAC + IR groups (resp., *P* = 0.006, *P* = 0.003). The difference between the scores of the RIP + IR and RIP + NAC + IR groups was not significant (*P* = 0.334) (Table 1).

**Figure 1:**
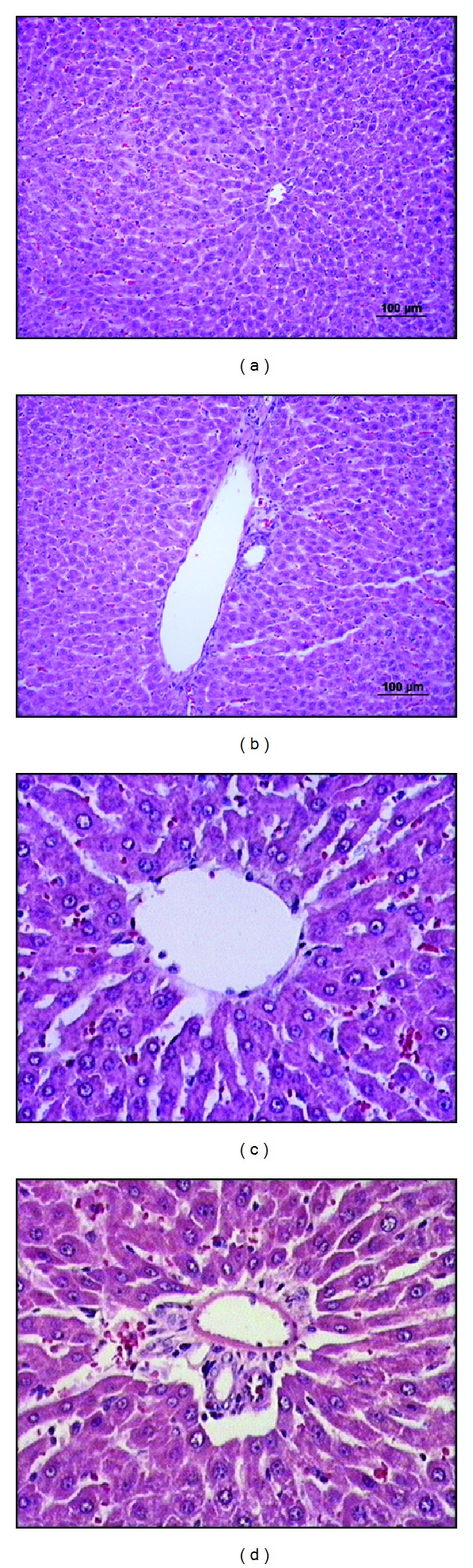
The liver sections of sham group (×20 and ×40). ((a) and (c)) normal sight of vena centralis and ((b) and (d)) normal sight of portal region.

In the sham group, normal morphological features were observed. Overall injury grade was 0 (Figures 1(a), 1(b), 1(c), and 1(d)).

**Figure 2:**
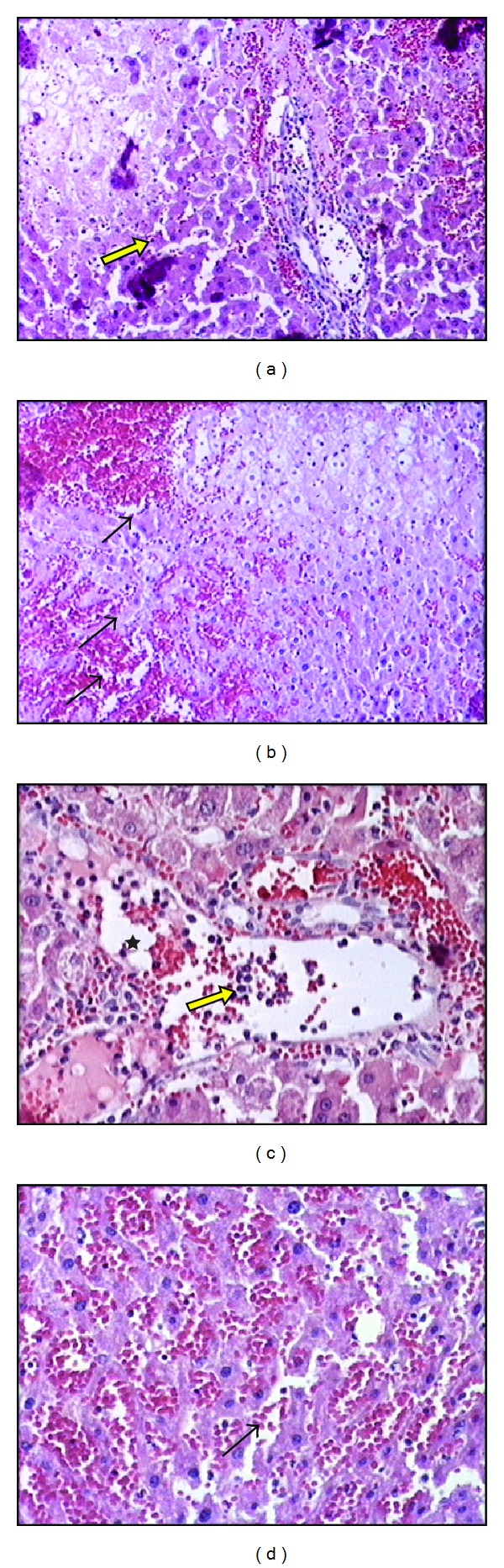
The liver sections of ischemia reperfusion group (×20 and ×40). ((a) and (c)) The integrity of hepatocyte cell cords is destroyed, sinusoidal dilatation, mononuclear cell infiltration (star), and focal necrosis of the hepatocytes (yellow arrows) ((b) and (d)). Erythrocyte extravasation (black arrows).

In the IR group, disintegration and hemorrhage in the hepatic chords, sinusoidal dilatation, and mononuclear cell infiltration was observed. In some regions focal necrosis were also detected (Figures 2(a), 2(b), 2(c), and 2(d)).

**Figure 3:**
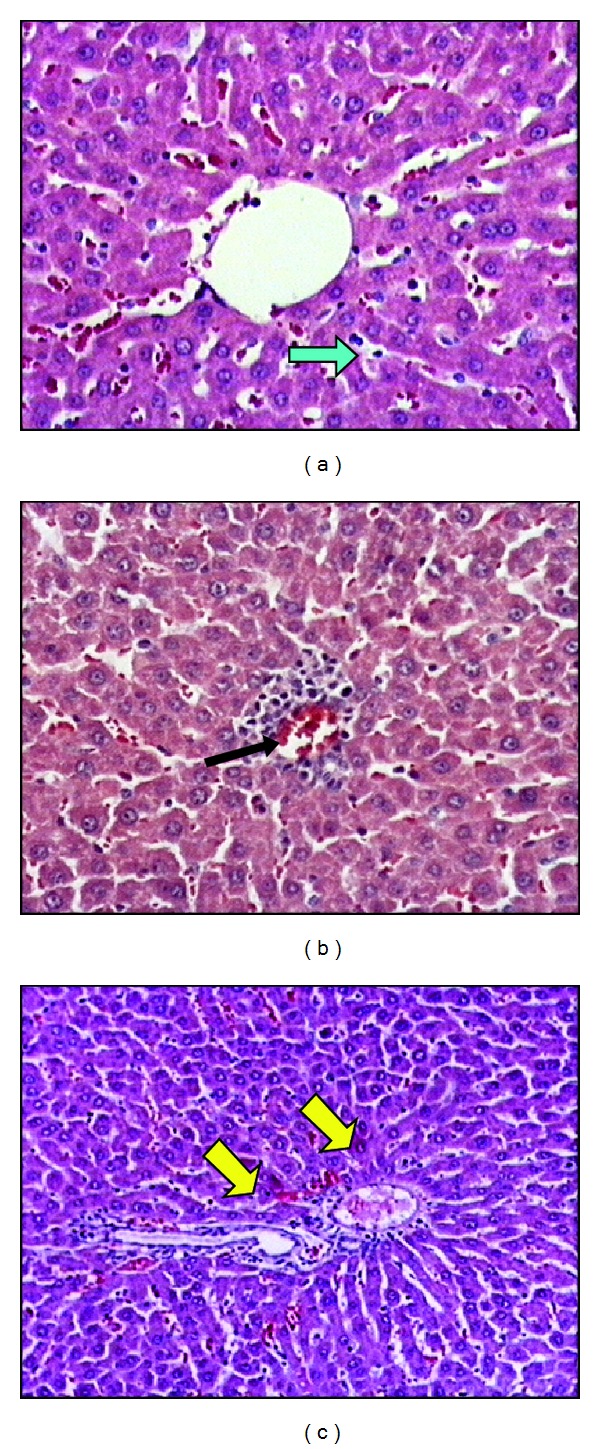
The liver sections of remote ischemic preconditioning + ischemia reperfusion group (×20 and ×40). (a) Blue arrow is showing the integrity of hepatocyte cell cords is more regular and less sinusoidal dilatation compared to ischemia reperfusion group. (b) Black arrow shows less focal necrosis. (c) Yellow arrows point the portal region which is close to normal.

In the RIP + IR group, integration of the hepatic chords was better and less sinusoidal dilatation, mononuclear cell infiltration, and degeneration of hepatic cells were observed compared to the IR group (Figures 3(a), 3(b), and 3(c)).

**Figure 4:**
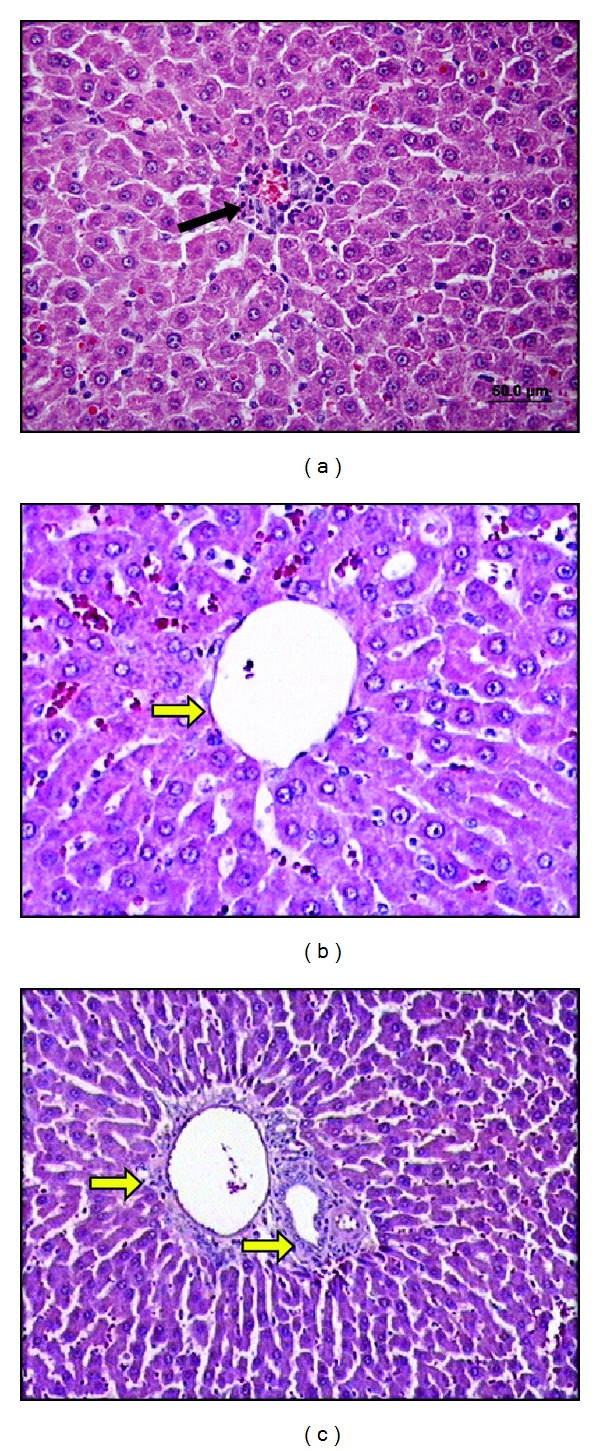
The liver sections of remote ischemic preconditioning + N-Acetylcysteine + ischemia reperfusion group (×20 and ×40). (a) Focal necrosis of the hepatocytes has been seen rarely (black arrow). ((b) and (c)) Less sinusoidal dilatation compared to ischemia reperfusion group and yellow arrows point the portal region which is close to normal.

In the RIP + NAC + IR group histopathological findings were similar to the RIP + IR group. Although no significant difference was observed, HHIS was lower than the RIP + IR group (Figures 4(a), 4(b), and 4(c)).

### 3.2. Biochemical Parameters

The values of AST and ALT for the IR (resp., *P* = 0.018, *P* = 0.018), RIP + IR (resp., *P* = 0.0003, *P* = 0.003), and RIP + NAC + IR (resp., *P* = 0.002, *P* = 0.002) groups were significantly higher than the sham group (Table 1). No statistically significant differences were determined for the values of AST and ALT in comparisons between the IR group and RIP + IR group (resp., *P* = 0.886, *P* = 0.086) and between the IR group and RIP + NAC + IR group (resp., *P* = 0.406, *P* = 0.064). Also there were no significant differences between the AST and ALT values of the RIP + IR and RIP + NAC + IR groups (resp., *P* = 0.775, *P* = 0.475).

## 4. Discussion

In the present study, it was demonstrated histopathologically that RIP and RIP + NAC decreased hepatic IR injury significantly. Although HHIS was better in the RIP + NAC group, there was no significant difference between RIP + IR and RIP + NAC + IR groups. According to the biochemical parameters, both methods could not prevent IR injury.

Ischemia/reperfusion injury induces cholestasis and reduces bile secretion temporarily. The changes of the bile flow result with an increase in AST/ALT levels, liver myeloperoxidase (MPO) activity, and plasma bilirubin levels and return to normal in 1–3 days [21]. The best indicators of hepatic IRI are enzyme activities like plasma AST, ALT, and histopathologic changes [6, 22]. Therefore we choose AST, ALT, and HHIS for detecting IRI.

Total [23] or partial [7] hepatic ischemia models can be used for hepatic IR studies. Partial ischemia causes less mesenteric congestion but besides its technical difficulty this model does not reflect the clinical practice. The total ischemia time that hepatic IRI can occur but does not impair the hemodynamic stability was determined as at least 25 minutes [19, 23]. In this study, 30 minutes of total hepatic ischemia was used because of the similarity to the clinical practice (Pringle manoeuvre).

In the present study hepatic IRI has been shown after 30 minutes of total hepatic ischemia with both biochemical and histopathological methods. Histopathological and biochemical changes in the IR group compared to the sham operated group indicated that hepatic IR model was applied correctly.

Remote IP was first described by Przyklenk et al. [24] who showed that brief occlusion of coroner arteries protects the heart against prolonged ischemia. The protective effect of RIP was also shown in lung [7], kidney [25], muscle [26], and bowel [27] IRI. RIP was suggested to be an effective and easy method for protecting the liver against IRI without generating a direct trauma to the liver [23, 28]. Lai et al. [28] showed that 4 cycles of IP by clamping the femoral artery before partial ischemia of the liver were effective to reduce the hepatic IRI. Küntscher et al. [18] reported that using a tourniquet would be as effective as direct clamping of the femoral artery for generating RIP. Abu-Amara et al. [29] showed that six cycles of RIP for four minutes before hepatic IR significantly reduced the hepatic IRI. Saita et al. [26] found that the most effective IP method is 10 minutes ischemia and 10 minutes reperfusion for 3 cycles for skeleton muscle IRI. Kanoria et al. [23] demonstrated that 3 cycles of RIP significantly reduce plasma levels of transferases and increase hepatic blood flow and peripheral oxygen saturation in a total hepatic ischemia model. Similarly, Şahin et al. [19] reported that 3 cycles of 10 minutes hind limb RIP protect liver from IRI biochemically and histopathologically. In the present study, 3 cycles of hind limb RIP, whose effectiveness was shown, were used but the protective effect of RIP could only be detected histopathologically.

N-Acetylcysteine participates in the glutathione syntheses in the lung and liver as a cysteine source and increases glutathione syntheses and it also bounds free oxygen radicals and protects the cell by preventing cell injury [9]. The hypothesis of this study was combination of RIP and NAC would provide a better tissue protection. This method was chosen because this technique could be easily performed before liver resection or transplantation.

The administration of NAC alone can provide tissue protection [13–17]. In the study of Smyrniotis et al. [16] it was shown that NAC (0.3 mg/kg IV) can reduce hepatic IRI biochemically and histopathologically. Galhardo et al. [14] found that 150 mg/kg IV NAC administration to rats before hepatic ischemia, reduced necrosis, apoptosis, and microvesicular steatosis compared to IR group. But controversial to these findings Ghosh et al. [30] and Baumann et al. [31] could not show the beneficial effects of 150 mg/kg IV NAC before hepatic ischemia.

In the present study we found that NAC (150 mg/kg IP) combination with RIP before hepatic total ischemia significantly decreased the HHIS compared to IR group. The HHIS of RIP + NAC group was lower than RIP but there was no statistically significant difference between these groups.

We could not confirm the beneficial histological effects of RIP and RIP + NAC with biochemical parameters that show the hepatic functions. Kanoria et al. [32] studied the effects of RIP, 3 cycles of 10 minutes, after 25 minutes of total hepatic ischemia and two hours of reperfusion histologically and biochemically. Unlike our results, they reported a significant reduction in plasma transferase levels and they were compatible with the histological findings. Likewise, Wang et al. [33] detected a significant reduction in ALT levels with RIP after hepatic IR at the first and third hours of reperfusion in mice. But the researchers could not find a significant effect at the second hour of reperfusion. We could not explain the biochemical results that did not confirm our histological findings but the difference of the study animals could be the reason of our varied results. Besides in this study only the early periods of reperfusion are investigated. The possible long-term effects of the drugs could be masked. Therefore newer studies that investigate long-term effects in different animal types are needed.

In conclusion, this study demonstrated that RIP and NAC addition to RIP decreased significantly hepatic IRI histopathologically.

## Figures and Tables

**Table 1:** Aspartate aminotransferase, alanine aminotransferase, and hepatic histopathological injury score values for the study groups.

	AST (U/L)	ALT (U/L)	HHIS
Sham group	138.4 ± 30.0	50.0 ± 9.7	0.00 ± 0.00
IR group	1034.7 ± 521.0*	834.2 ± 427.6*	2.28 ± 0.48*
RIP + IR group	1081.8 ± 227.5*	1203.6 ± 297.8*	1.28 ± 0.48^∗#^
RIP + NAC + IR group	1703.8 ± 1145.3*	1237.4 ± 540.5*	1.00 ± 0.57^∗#^

*P < 0.05, compared to sham group.

^#^ *P* < 0.05, compared to IR group.

AST: aspartate aminotransferase, ALT: alanine aminotransferase, HHIS: hepatic histopathological injury score, IR: ischemia-reperfusion, RIP: remote ischemic preconditioning, NAC: N-Acetylcysteine.
